# Relevance of *Leptospira* in boar and for the development of alternative antimicrobial concepts in boar semen preservation

**DOI:** 10.1186/s40813-020-00169-9

**Published:** 2020-11-18

**Authors:** Kathi Scheinpflug, Sabine Schiller, Helen Jäkel, Martin Schulze, Dagmar Waberski, Kristin Mühldorfer

**Affiliations:** 1 Leibniz Institute for Zoo and Wildlife Research, Alfred-Kowalke-Str. 17, D-10315 Berlin, Germany; 2 Unit for Reproductive Medicine of the Clinics/Clinic for Pigs and Small Ruminants, University of Veterinary Medicine Hannover, Bünteweg 15, D-30559 Hannover, Germany; 3 Institute for Reproduction of Farm Animals Schönow, Bernauer Allee 10, D-16321 Bernau, Germany

**Keywords:** Antibiotics, Artificial insemination, Leptospires, Leptospirosis, Boar, Venereal diseases

## Abstract

Leptospirosis is a zoonotic disease of importance to public health and in livestock productions. It causes significant economic losses in pig breeding farms worldwide. However, actual transmission cycles and disease epidemiology in the pig population remain largely unknown. Despite the fact that the potential risk of venereal transmission of pathogenic *Leptospira* serovars in pigs has been a topic of discussion since the 1970s, reliable data are still lacking compared to other livestock species. Consequently, antibiotics are added to semen extenders to reduce bacterial contamination including pathogens like *Leptospira*. In view of the global threat of antimicrobial resistances, the routine use of antibiotics in porcine semen extenders is now under debate. Information about the prevalence of *Leptospira* infections in boar used for artificial insemination is needed for the development of novel antimicrobial concepts in pig insemination.

This short report provides a summary of the state of knowledge, together with negative results from real-time PCR analyses for the detection of pathogenic *Leptospira* DNA in boar semen. Molecular analyses were performed on 96 raw and extended samples obtained from normospermic ejaculates of 58 boar housed in six different studs in Germany. In the absence of reliable data, it is important to raise the awareness for a subject that can represent a challenge for pig productions in keeping reproductive health and food safety at high levels. The present molecular results indicate that *Leptospira* might not be a common threat in boar semen. Conclusive evidence would require results from a systematic serological surveillance of boar, combined with seasonal molecular analyses of semen to identify potential carriers, and assess actual seroprevalences, associated *Leptospira* serovars and transmission events.

## Introduction

Artificial insemination (AI) has become the most important biotechnology in pig reproduction. It is already used for more than 90% of sows in breeding farms [1] in order to disseminate favorable genetic traits, while minimizing potential risks for venereal infections [2]. *Leptospira* are common bacterial pathogens of urogenital tract infections in animals and can persist in the kidneys of infected hosts [3]. They may also persist in the reproductive organs as shown for cattle [4] and pigs [5, 6], although associated studies in domestic boar are sparse [6, 7]. The possibility, that leptospires could be present in semen and subsequently transmitted by AI, has been investigated by many authors with ambiguous results. Strong evidence has been reported for cattle, small ruminants and horses based on molecular detection of *Leptospira* DNA in semen samples (e.g., [8–10]). For pigs, however, reliable data are still lacking and the actual transmission risk through AI remains unclear [2, 11–13].

Potential transmission routes for porcine *Leptospira* infections, including venereal transmission and associated studies from the 1960s onwards, have already been summarized and discussed by Bolt in 1990 [14]. More recent studies from Vietnam [15, 16], Brazil [17], Kenya [18] and Germany [19] focused on seroprevalences of sows and potential risk factors in pig husbandry. The authors consistently reported on varying serovar-dependent but predominantly high *Leptospira* seroprevalences in sows. Most common *Leptospira* serovars belong to the serogroups Australis, Icterohaemorrhagiae, Autumnalis and Pomona. Potential sources of infections are infected sows and other domestic animals as well as insufficient prophylactic measures, such as inadequate rodent control, introduction of pig carriers, absence of a quarantine regime and/or vaccination, and deficient hygiene measures. Risk analyses from studies in Vietnam and Brazil showed a potential association of serovars Pomona [15], Icterohaemorrhagiae and Castellonis [17] with the reproduction regime (AI vs. natural mating or both), indicating a higher prevalence in farms that only use AI for breeding. Venereal transmission was already assumed for serovars Bratislava and Pomona as an important route for porcine infections due to their persistence in the genital tract and detection in genital fluids [5–7]. However, an experimental infection of boar with *Leptospira* Pomona did not result in venereal transmission to sows via natural mating, even though the boar were leptospiruric [20].

As a precaution, antibiotics are routinely added to semen extenders to reduce the general risk of bacterial contamination including pathogenic *Leptospira*, which is implemented in the Council Directive 90/429/EEC (2012), Annex C, of the European Union. The global increase of antimicrobial resistances, however, demands the development of alternative strategies for semen preservation [21]. Recently, we proposed novel antimicrobial concepts in AI of pigs for removal ([22], Jäkel et al. in revision) or replacement [23, 24] of conventional antibiotics. These concepts have shown efficiency against commensal and opportunistic bacteria usually occurring in boar ejaculates. There is debate in AI practice as to whether leptospires need particular attention, as they demand special growth conditions and are not included in microbiological screenings of boar semen for quality control. To answer this question it is important to gain information about the relevance of *Leptospira* infections in domestic boar used in AI and the actual transmission risk through semen.

In the present study we investigated the presence of *Leptospira* DNA in boar semen using a validated realtime PCR analysis with a proven detection range and sensitivity for pathogenic serovars. This molecular approach is a first step to clarify whether *Leptospira* need to be specifically targeted by future antimicrobial concepts in boar semen preservation.

## Materials and methods

This study was part of a joint research project that aims for the development of a feasible low-temperature storage concept for liquid, antibiotic-free preservation of boar semen [22, 24, 25]. Molecular analyses were performed for the detection of pathogenic *Leptospira* DNA in 96 semen samples collected in 2018 and 2019 from 58 healthy, mature and fertile boar in Germany. Forty-nine animals originated from five different AI centers (boar stud 1 to 5) and nine animals were kept in a university livestock husbandry (boar stud 6; details in Table 1). All boar were routinely used for the production of AI doses with 2 to 5 days of rest between semen collections. They received commercial feed pellets for AI boar and were housed in individual pens (2 × 3 m) with straw bedding or sawdust litter, equipped with nipple drinkers according to the European Commission Directive for Pig Welfare. Boar were dewormed twice a year and vaccinated against swine erysipelas and parvovirus. Rodent control was carried out in all AI centers.

**Table 1:** Characteristics of boar studs and semen samples used for molecular analysis

Boar stud	Stud size	Bedding type	Replacement rate of boar	Sampling time	Sampled boar	Age of boar	Raw semen	Diluted semen
					n	months	n	n
1	290	sawdust	55%	11/2018	10	19.9 ± 3.1	none	10
2	450	sawdust	60%	12/2018	10	17.9 ± 4.4	none	10
3	300	straw	50%	01/2019	9	16.9 ± 4.5	9	9
4	100	straw	45%	02/2019	10	17.7 ± 7.9	10	10
5	330	sawdust	60%	03/2019	10	23.3 ± 6.4	10	10
6	10	straw	40%	06/2018	9	36.0 ± 18.0	9	9

Stud size refers to the number of boar kept

Diluted semen originated from ejaculates of the same boar investigated

The 96 samples consisted of 38 normospermic ejaculates (raw semen), 38 extended semen portions of the same ejaculates, and 20 extended semen portions from other boar (one per boar) where raw semen was not available (Table 1). Semen samples (*n* = 58) were extended in AndroStar® Premium (Minitüb, Germany) without antibiotics; processing details are described in Hensel et al. [24] and Jäckel et al. (in revision). Both sample types (raw and extended semen) were included in the analysis to control DNA extractions and PCR results for potential positive and negative dilution-associated effects on PCR performance and sensitivity, resulting from a high content of lipid-rich sperm cells and host DNA in raw semen as well as from a decrease of pathogen DNA in extended semen, respectively.

DNA was extracted from 300 μl per sample, starting with a prewash step, as stated in the *Current Protocols in Molecular Biology* for “Preparation of genomic DNA from mammalian sperm” [26] and instructions from the DNeasy Blood & Tissue kit (Qiagen, Germany) for purification of DNA from nails, hair or feathers. A real-time PCR targeting the LipL32 gene was performed by using the well-established protocol, primers and probe from Stoddard et al. [27] for the detection of pathogenic *Leptospira* and the SsoAdvanced™ PCR Supermix (Bio-Rad, Germany). The analysis was carried out using the Stratagene Mx3005P system (Agilent Technology, Germany). DNA from a laboratory strain of *Leptospira kirschneri* serovar Grippotyphosa was used as positive control, which was kindly provided by the consultant laboratory for *Leptospira* of the Federal Institute for Risk Assessment in Berlin, Germany.

## Results and discussion

All 96 semen samples were negative for pathogenic *Leptospira* DNA in the current real-time PCR analyses, including serovars present in the pig population. Porcine leptospirosis is a largely unknown zoonotic disease of public health and economic importance [12, 19]. It belongs to the reportable epizootics in Germany. To our knowledge, there is no clear uniform scope of action or implemented quality system in AI centers within the European Union and most probably worldwide for serological tests, a comparable serovar panel and interpretation of results. Thus, the present study stimulates further research in this area. Given that boar are usually kept under strict hygienic measures and only enter a stud after passing quarantine, the risk for the occurrence of *Leptospira* infections in AI boar seems to be low. The importance of appropriate control measures and dry, temperature controlled housing in boar husbandry is however strengthened by the fact that rodents act as primary reservoirs for pathogenic leptospires and that the bacteria survive well in warm, moist environments. Moreover, subclinically infected boar could bear a potential risk for shedding *Leptospira* in their semen. In the present study, *Leptospira*-DNA was absent in semen samples although the boar can be infected. The incorporation of routine serological tests in sanitary guidelines for AI centers could therefore facilitate the replacement of serological positive or suspicious carriers with those of healthy individuals of proven semen quality and fertility [28]. Although practicability and economic impact would need to be considered, these measures would facilitate the omission of *Leptospira*-specific antibiotics in semen extenders and therefore support the global antimicrobial resistance defense strategy.

## Conclusion

With negative molecular results from this study we want to challenge discussions as to whether boar semen pose a serious risk for the transmission of pathogenic *Leptospira*. In the absence of reliable data, large scale studies in different countries are encouraged to assess actual seroprevalences in boar and the occurrence of specific *Leptospira* serovars that should be considered for venereal infections. The knowledge would clearly enhance our understanding of the epidemiology of *Leptospira* infections in pig productions and largely influence the development of alternative strategies to the currently used conventional antibiotics in semen extenders.

## Data Availability

The dataset generated in the current study is available from the corresponding author on reasonable request.

## Abbreviations

AI: Artificial insemination
